# The Aqueous Extract from *Ceratonia siliqua* Leaves Protects against 6-Hydroxydopamine in Zebrafish: Understanding the Underlying Mechanism

**DOI:** 10.3390/antiox9040304

**Published:** 2020-04-08

**Authors:** Sara Abidar, Razvan Stefan Boiangiu, Gabriela Dumitru, Elena Todirascu-Ciornea, Amina Amakran, Oana Cioanca, Lucian Hritcu, Mohamed Nhiri

**Affiliations:** 1 Laboratoire de Biochimie et Génétique Moléculaire, Faculté des Sciences et Techniques, Université Abdelmalek Essaadi, Tanger Principal BP 416, Morocco; sara.abidar91@gmail.com (S.A.); amakran_amina@hotmail.com (A.A.); med.nhiri@gmail.com (M.N.); 2 Department of Biology, Faculty of Biology, Alexandru Ioan Cuza University of Iasi, 700506 Iasi, Romania; boiangiu.razvan@yahoo.com (R.S.B.); ciornea@uaic.ro (E.T.-C.); 3 Department of Pharmacognosy, Faculty of Pharmacy, “Grigore T. Popa” University of Medicine and Pharmacy, 16 University Street, 700115 Iasi, Romania; oana.cioanca@gmail.com

**Keywords:** *Ceratonia siliqua*, aqueous extract, 6-hydroxydopamine, memory, oxidative stress

## Abstract

*Ceratonia siliqua* L. is a Mediterranean medicinal plant traditionally cultivated for its ethnopharmacological benefits, such as antidiarrheal, antidiabetic, enhance acetylcholine, antioxidant, antiatherosclerotic, and for its possible anti-neurodegenerative potential. The aim of the present study was to evaluate the chemical composition, as well as the cognitive-enhancing, anxiolytic, and antioxidant activities of the aqueous extract from *C. siliqua* (CsAE) leaves against 6-hydroxydopamine (6-OHDA) zebrafish Parkinson’s disease (PD) model. CsAE (0.1, 0.3, and 1 mg/L) was administered by immersion to zebrafish (*Danio rerio*) for eight consecutive days and one hour before each behavioral test of each day, while 6-OHDA (250 µM) treatment was supplied one day before the novel tank diving test (NTT). Qualitative and quantitative analyses were performed by the ultra-high-performance liquid chromatography (UHPLC) analysis. The memory performance was evaluated through the NTT and Y-maze tests. Additionally, the *in vitro* and *in vivo* antioxidant status and acetylcholinesterase (AChE) activity was also assessed. Our finds demonstrated that CsAE presented positive antioxidant and anti-AChE activities, which contributed to the improvement of cognitive function in the 6-OHDA zebrafish PD model.

## 1. Introduction

Parkinson’s disease (PD) represents the other common neurodegenerative disease after Alzheimer’s disease (AD) [1]. PD is characterized by tremor, rigidity, bradykinesia [2], and postural instability [3]. These symptoms are mainly attributed to the dopaminergic neurons depletion in the *substantia nigra pars compacta* [4], leading to a subsequent loss of dopamine in the striatum [5]. PD is accompanied by a presence of eosinophilic and intracytoplasmic inclusions called Lewy bodies [6]. There is now considerable evidence showing that the degeneration also affects the cholinergic [7], noradrenergic [1], adrenergic, and serotoninergic systems [6] resulting in non-motor symptoms namely sleep disorders [8], cognitive problems ranging from memory impairment to dementia [7], depression, anxiety, psychosis and apathy, all affecting patients life quality [9]. 

With the high prevalence and the absence of treatment to curate PD [10], several toxin models are provided to understand the pathogenesis and to develop new strategies against PD [11]. Among the most recognized PD models, the 6-hydroxydopamine (6-OHDA), a neurotoxin inducing depletion of dopaminergic neurons in the *substantia nigra pars compacta* [12]. It has experimented in cell cultures [13] in rodents, in which the injection is localized in the medial forebrain bundle or other parts of the dopaminergic pathway [14] and zebrafish [13]. Used as a model organism of vertebrate development [15], nowadays, zebrafish presents an encouraging model of neurodegenerative disorders such as AD, PD, Huntington disease, and schizophrenia [16] due to its neuroanatomical and neurochemical pathways similitudes with the human [17], and its high fecundity and short generation time allowing the evaluation of large drug varieties [13]. 

Reactive oxygen species (ROS) are molecules generated during the normal cellular metabolism in aerobic life [18] and containing one or more than an unpaired electron in one atomic orbital, giving them a high reactivity degree [19]. The excess production of ROS, accompanied by the insufficient antioxidative defense, is described as oxidative stress [20]. Numerous studies have shown that oxidative stress is correlated with the pathophysiology of PD [21]. Indeed, the brain is more vulnerable to attacks from free radicals leading to neurodegeneration: on the one hand, the brain consumes more oxygen under physiological conditions, but then, it contains fewer amounts of endogenous antioxidant compounds [22]. Many lines of evidence prove this hypothesis: the high amounts of ROS detected in particular brain areas [23], the increased levels of free iron accumulated in the brain during PD [3], and the presence of lipid peroxidation markers such as the 4-hydroxynonenal and malondialdehyde (MDA) in the *substantia nigra* of PD patients [19]. Furthermore, the superoxide dismutase (SOD), catalase (CAT), peroxidase, and glutathione (GSH)—representing the endogenous antioxidative system—are found at lower levels in the *substantia nigra* of PD patients [24]. 

Phenolic compounds are metabolites derived from L-phenylalanine [25], including a large group of substances such as phenolic acids, hydroxycinnamic acids, lignans, tannins, and flavonoids [26]. Phenolic compounds improve color, flavor, and food quality [27]. They have shown beneficial effects on human health [28], suggesting their use in pharmaceutical industries [29]. Indeed, they possess antioxidant activity [30], inhibiting, thus, ROS [31], such as superoxide anion (O_2_^−^), hydroxyl radical (OH^−^), and hydrogen peroxide (H_2_O_2_) [32], contributing to the prevention of several diseases including cancer, cardiovascular illnesses [27] and neurodegenerative diseases [33]. These natural compounds will constitute an alternative to the use of synthetic antioxidants (tertiary butyl hydroxy quinone (TBHQ), butylated hydroxyanisole (BHA), and propyl gallate (PG)), which have already shown carcinogenic effects [34]. 

*Ceratonia siliqua* L. (*C. siliqua* L.) or carob, belongs to the family of Leguminosae [35] and the sub-family of Caesalpinaceae [36]. It is widespread in the Mediterranean basin [37]. Carob is a perennial tree, evergreen [36], dioecious with some hermaphrodite forms [27]. *C. siliqua* L., naturally grown in arid regions [38], and is a salinity and drought-resistant plant [27,35]. In Morocco, it is distributed in the hems of the Atlas chains, in the Rif Mountains and in some southwestern valleys where the climate is arid and semiarid [39]. *C. siliqua* L. is rich in A, B vitamins, insoluble fibers, sugar, and other minerals [38,40]. *C. siliqua* L. is one of the most valuable plants as a source of gallic acid [41], catechol, pyrogallol, and chlorogenic acid [42]. Nowadays, economic gain [43] has been experienced by various sectors such as industrial [34], ornament [39], cosmetics [37], and health [28]. Different parts of *C. siliqua* L. (leaf, flower, fruit, wood, bark, and root) are used in Moroccan pharmacopeia [43]. There have been very few studies that have evaluated the antioxidant activity of leaves from this species [27,44]. For this reason, the aqueous extract from *C. siliqua* L. (CsAE) leaves from southern Morocco was screened about its total phenolic, flavonoids, and condensed tannins contents. The *in vitro* antioxidant activity was measured through three tests: 2,2-diphenyl-1-picrylhydrazyl (DPPH) scavenging assay, ferric reducing power, and iron-chelating ability. Furthermore, the *in vitro* assay of acetylcholinesterase (AChE) inhibition was also carried out. The ultra-high-performance liquid chromatography (UHPLC) semiquantitative analysis was used to determine the main phenolic compounds from CsAE. In the second set of analyses, CsAE was also investigated for its eventual capacity to abolish anxiety, memory deficits, brain oxidative stress and to inhibit AChE in a 6-OHDA zebrafish model of PD.

## 2. Materials and Methods 

### 2.1. Plant Material

The leaves of *C. siliqua* L. were harvested in August 2017 from spontaneous trees in the south of Morocco (Tafraoute ). The collected samples were identified by Professor Mohamed Kadiri (Laboratory of Diversity and Conservation of Biological Systems, Department of Biology, Faculty of Science of Tetouan, Morocco). The samples were washed with tap water and distilled water, dried in the oven at 40 °C, and then ground using an electric grinder to obtain a powder, which was stored at 4 °C before analyses. 

### 2.2. Extraction 

CsAE was prepared according to Chen et al. [45] with some modifications: 10 g of the plant powder was mixed with 500 mL of distilled water and incubated at 80 °C for 20 min; the mixture was centrifuged at 3000 rpm for 10 min at 25 °C. The supernatants were recovered and placed into the rotavapor. The dry matter yield was determined through the equation: DMY (%) = (Me/Mp) × 100, where DMY (%) = Dry matter yield, Me = Mass of the extract after the evaporation of the solvent and Mp = Mass of the plant used for the extraction. CsAE showed 93% of DMY. 

### 2.3. Total Phenolic Content 

The total phenolic content (TPC) of the CsAE was determined by the Folin–Ciocalteu method with some modifications [46]: in a 96-well microtitre plate, 20 µL of diluted sample was mixed with 100 µL of Folin–Ciocalteu reagent (prepared by the mixture of 1 mL of Folin–Ciocalteu reagent and 9 mL of distilled water). After 4 min, 80 µL of saturated sodium carbonate solution (75 g/L) was added. After 30 min of incubation at room temperature, the absorbance at 765 nm was measured. Gallic acid hydrate at different concentrations was used as standard. TPC was estimated using the following linear equation: A = 0.191C−0.030; *R^2^* = 0.999, where A is the absorbance, C is the total phenolic content expressed as gallic acid equivalents (GAE) per dry weight (DW) of plant material (mg GAE/g DW).

### 2.4. Flavonoids Content

The flavonoids content (FC) was estimated using a 96-well microplate plate, following the aluminum chloride (AlCl_3_) method, with some modifications [18]. Firstly, 10 µL of potassium acetate (1 M), then 80 µL of distilled water and finally 10 µL of AlCl_3_ (10%) were added to 25 µL of diluted extract or fraction and 75 µL of absolute methanol. The mixture was incubated in obscurity at room temperature for 30 min; the absorbance was measured at 415 nm using a spectrophotometer. Quercetin at different concentrations was used as the reference standard. FC was calculated using the linear equation: A = 0,150C−0.133; *R^2^* = 0.994 where A is the absorbance, C is the flavonoid content expressed as quercetin equivalents (QE) per dry weight (DW) of plant material (mg QE/g DW).

### 2.5. Condensed Tannins Content

The condensed tannins content (CTC) was estimated by the Folin–Denis method, according to Kusirisin et al. [47], with some modifications. The reaction was carried out in a 96-well microplate reader. The mixture contained 15 µL of the sample, 25 µL of Folin–Denis reagent, 50 µL of Na_2_CO_3_ (0.5%) and 100 µL of distilled water. The mixture was vigorously stirred and then incubated at room temperature for 30 min. The absorbance was measured at 775 nm. The standard was prepared using different concentrations of tannic acid. Results were expressed as tannic acid equivalents (TAE) per dry weight (DW) of plant material (mg TAE/g DW) using the following equation: A = 0.180C−0.151; *R*^2^ = 0.992, where A is the absorbance and C is the condensed tannins concentration.

### 2.6. UHPLC Semiquantitative Analysis

The sample analysis was performed using an UltiMate 3000 Thermo Fisher system, in a gradient of A (acetonitrile with 0.1% phosphoric acid) in B (0.1% phosphoric acid) as follows: 0–4 min. 10–15%, the next 4 min isocratic at 15%, 8–15 min 30%, 15–18 min 40%, 18–22 min 55%, then for the next 3 min, return to initial conditions. The column used for separation of the compounds was Luna Omega 5 µ Polar C18 (100A, 150 × 4.6 mm). The sample injection was 2 µL in a flow of 0.8 mL. Detection was assessed at seven different band wavelengths between 220 nm and 800 nm, taking into consideration 245 nm, 280 nm, 330 nm, and 521 nm; these four detection bands represent the maximum absorption bands for flavonoids, phenolic acids, and anthocyanidins respectively. Each peak was identified using the UV spectra and the library data available and was compared to the standards. Calibration curves were obtained for several standards (gallic acid, luteolin-7-O-glucoside, quercetin-3-β-glucoside, epicatechin, cyanidin-glucoside, rosmarinic acid), which were used for the quantification of the identified compounds.

### 2.7. Antioxidant Activity Assays

The DPPH scavenging assay was evaluated according to Velazquez et al. [48], with minor modifications using a 96-well microplate reader: 150 µL of DPPH^.^ solution (4 mg of DPPH diluted in 100 mL of absolute methanol) was incubated with 50 µL of the diluted sample at room temperature in obscurity for 30 min. Absorbance was then measured at 517 nm. The same procedure was repeated with ascorbic acid as the positive control. The negative control contained DPPH and methanol. The blank was the methanol. 

Ferric reducing antioxidant power (FRAP) of extracts and fractions was evaluated via the test based on the capacity to reduce Fe^3+^ (FeCl_3_) to Fe^2+^ (FeCl_2_), according to Chahmi et al. [49], with some modifications. Briefly, 125 µL of each sample was mixed with 313 µL of phosphate buffer sodium (0.2 M; pH 6.6) and 313 µL of K_3_Fe(CN)_6_ (1%). After 10 min of incubation at 50 °C, 313 µL of trichloroacetic acid (10%) was added, then 313 µL of distilled water and 66 µL of FeCl_3_ (0.1%). The absorbance was measured at 700 nm. The blank contained all the reagents except the sample, which was substituted by distilled water. Butylated hydroxytoluene (BHT) at different concentrations was used as standard.

The iron chelating activity of extracts and fractions was estimated using a 96-well microplate reader, via the protocol of Meghashri et al. [50] with some modifications: 50 µL of the sample was mixed with 25 µL of FeCl_2_ (2 mM) and 58.3 µL of absolute methanol, the reaction was initiated with the addition of 25 µL of ferrozine (5 mM). The mixture was vigorously stirred and incubated at room temperature in obscurity for 10 min. The absorbance was measured at 562 nm. Absolute methanol was used as the blank of the reaction. The negative control was the reaction mixture except for the sample, which was substituted by methanol. The ethylenediaminetetraacetic acid (EDTA) was a positive control.

### 2.8. In Vitro Inhibition Assay of Acetylcholinesterase 

The inhibition test of acetylcholinesterase (AChE) was determined through the Ellman’s method with minor modifications [51], 50µL of Tris–HCl buffer (50 mM; pH 8.0) added to 25 µL of acetylthiocholine iodide (ATCI) and 125µL DTNB (3 mM) and 10 µL of CsAE. All the reagents were mixed vigorously, then, 25 µL of AChE (0.25 U/mL). Absorbances were measured at 415 nm.

Results were expressed as the following equation: Inhibition (%) = A_control_ − A_sample_/A_control_.

Control is the mixture with methanol instead of the extract. Galanthamine hydrobromide was used as a positive control; it was tested at concentrations 15, 30, 60, 125, and 250 µg/mL.

All experiments were carried out in triplicate (*n* = 3); data are expressed as mean ± standard error. The IC_50_ values (the concentration of test compounds that inhibits hydrolysis of substrates by 50%) were determined by spectrophotometric measurement of the effect of increasing test compound concentrations (CsAE and galantamine hydrobromide) on AChE activity. The IC_50_ values were obtained from dose-effect curves by linear regression. 

### 2.9. Animals 

**Figure 1:**
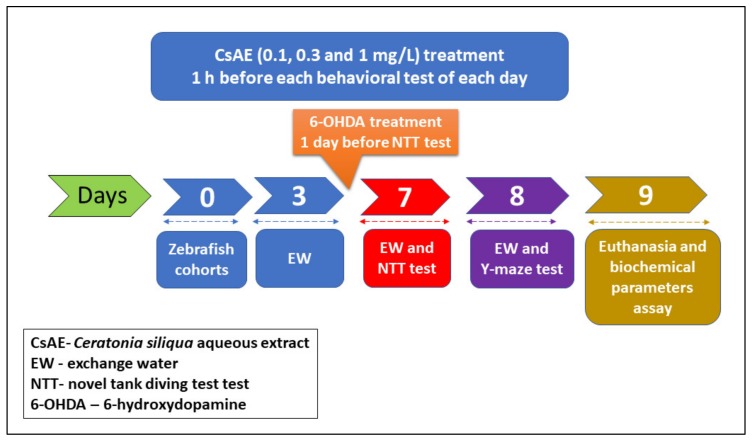
Schematic representation of the experimental protocol.

Fifty adults zebrafish (*Danio rerio*) of wild-type short-fin strain (sex ratio: 50:50 male: female, 3-4-month-old, and 3–4 cm in length) purchased from an authorized commercial dealer (Pet Product S.R.L., Bucharest, Romania) was used in the present study. Fish were randomly divided into groups of 10 fish/24 L housing tanks filled with unchlorinated water, equipped with constant aeration and a light-dark cycle (14/10 h) photoperiod (lights on at 8:00 am). Water temperature within the tanks was maintained at 26 ± 1 °C; pH 7.5; dissolved oxygen at 7.20 mg/L; ammonium concentration <0.004 ppm; conductivity 500 µS and filtrated to avoid the accumulation of organic toxins. Fish were fed twice daily with Norwin Norvitall flake (Norwin, Gadstrup, Denmark). For the behavioral studies, zebrafish were kept for 14 days to accommodate in laboratory conditions and then were divided into 5 groups: the control, the 6-hydroxydopamine (6-OHDA, 250 µM, Sigma-Aldrich, Darmstadt, Germany), and three CsAE treatment groups (0.1, 0.3, and 1 mg/L) administered in three consecutive steps, from 0 to 7 days. The doses of 6-OHDA and CsAE were chosen according to previous reports [52,53]. CsAE (0.1, 0.3, and 1 mg/L) was individually delivered to fish through transferring into 500 mL glass for 1 h, once daily, whereas 6-OHDA (250 µM) treatment was once administered independently by moving into a 500 mL glass, one day before NTT test. Experimental protocol (as is summarized in Figure 1) was approved by the local board of ethics for animal experimentation (No. 15309/2019) and fully complied with the Directive 2010/63/EU of the European Parliament and of the Council of 22 September, 2010 on the protection of animals. Efforts were made to reduce animal suffering and the number of animals utilized. 

### 2.10. Behavioral Analysis

#### 2.10.1. Novel Tank Diving Test (NTT)

The NTT is a specific test used for assessing anxiety in zebrafish, as described by Cachat et al. [54]. The trapezoidal tank (1.5 L) used measured 15.2 height × 27.9 top × 22.5 bottom × 7.1 width cm, equally divided into two horizontal sections (top and bottom). After 1 h of CsAE treatment, the animals were placed individually within the test tank without acclimatization, and swimming behavior was recorded for 6 min. The animals were recorded with a Logitech HD Webcam C922 Pro Stream camera (Logitech, Lausanne, Switzerland) placed 30 cm away from the tank, and the videos were analyzed using ANY-Maze^®^ software (Stoelting CO, Wood Dale, IL, USA). The following parameters were registered: total distance in the tank (m) (to assess the locomotor activity), for the anxiety-like behavior: time spent in the top zone of the tank (s), and time spent in the bottom zone of the tank (s) were recorded. Representative tracking images of zebrafish locomotor activity from each group were obtained at the end of the analysis with ANY-Maze^®^ software. Imipramine (IMP, 20 mg/L, Sigma-Aldrich, Darmstadt, Germany) was used as the positive control in the NTT test. 

#### 2.10.2. Y-Maze Test

Spatial memory in zebrafish was assessed using the Y-maze task [55]. The apparatus consisted of a Y-maze glass aquarium with three arms (25 cm long, 8 cm wide and 15 cm high), filled with 3 L of the same water used in the home aquarium. After 1 h of CsAE treatment, each fish was tested individually during 5 min session. Donepezil (DP, 10 mg/L, Sigma-Aldrich, Darmstadt, Germany) was used as the positive control in the Y-maze test. The behavioral activity was analyzed using ANY-Maze^®^ software (Stoelting CO, Wood Dale, IL, USA) and with a Logitech HD Webcam C922 Pro Stream camera (Logitech, Lausanne, Switzerland) placed above the Y-maze tank. The following measures were recorded: the percent spontaneous alternation (to assess short-term spatial memory), and for the locomotory activity, the number of arm entries was recorded. Representative tracking images of zebrafish locomotor activity from each group were obtained at the end of the analysis with ANY-Maze^®^ software.

### 2.11. Biochemical Parameters Assay 

Immediately after behavioral tests, zebrafish were euthanized (10 min immersion in ice water, 2–4 °C) until loss of opercular motions [56], and their whole brains were isolated for biochemical parameters assay. The brains were gently homogenized in ice 0.1 M potassium phosphate buffer (pH 7.4), 1.15% KCl with Potter Homogenizer (Cole-Parmer, Vermon Hills, IL, USA). The resulted homogenate was centrifuged at 960× *g* for 15 min. The supernatant was used for the estimation of acetylcholinesterase (AChE), superoxide dismutase (SOD), catalase (CAT), glutathione peroxidase (GPX) specific activities, and malondialdehyde (MDA) level, following the methods already described in detail by Dumitru et al. [55]. Estimation of protein content was done through a bicinchoninic acid (BCA) protein assay kit (Sigma-Aldrich, Darmstadt, Germany) [57].

### 2.12. Statistical Analysis 

Results from three independent experiments are expressed as mean ± standard error of the mean (SEM). Results were statistically analyzed by one-way analysis of variance (ANOVA) followed by Tukey’s *post hoc* multiple comparison test, considering treatment as a factor. The difference showing a *p* level of 0.05 or lower was considered statistically significant. GraphPad Prism 8.0 (GraphPad Software, Inc., San Diego, CA, USA) was used to perform statistical analyses. Correlation between the behavioral scores, enzymatic activities, and lipid peroxidation was estimated by the Pearson correlation coefficient (*r*). 

## 3. Results and Discussion

### 3.1. Total Phenolic Content

The highest amount of TPC was recorded in CsAE with 52.95 ± 0.141 mg GAE/g DW, which agrees with the study of Uysal et al. [58], carried out on CsAE from Turkey. Thus, in the study of Hsouna et al. [40], the TPC in *C. siliqua* leaf crude extract/water fraction was 130 ± 5.62 mg GAE/g. Moreover, the TPC in *C. siliqua* aqueous extract was 54.2 mg GAE/g DW, as obtained by Alali et al. [59]. Othmen et al. [60] reported quantities of 28.67 ± 0.09 g GAE/100 g DW *C. siliqua* leaf aqueous extract for TPC. In contrast, Aboura et al. [61] reported that TPC of *C. siliqua* leaf infusion was 8.93 mg GAE/100 mL infusion. Based on these findings, CsAE exhibits a TPC comparable with those reported by other authors that assume its antioxidant and memory-facilitating activities. 

### 3.2. Flavonoids Content

Supporting evidence suggested that the intake of flavonoids have been associated with health benefits such as antioxidant and anti-inflammatory effects, increased neuronal signaling, and improved metabolic functions [62]. In our study, the value for FC from CsAE was 25.35 ± 0.124 mg QE/g DW. Othmen et al. [60] reported value for *C. siliqua* leaf aqueous extract of 1.43 g rutin equivalents (RE)/100 g DW for FC. Sassi et al. [63] reported that 1 mg of *C. siliqua* leaves aqueous extract contains 188 µg quercitin. Vaya and Mahmood [64] reported that the leading free flavonoid was myricetin (340 mg/kg extract) from *C. siliqua* extract. 

### 3.3. Condensed Tannins Content

Condensed tannins or proanthocyanidins have been demonstrated to prevent neuron damage, decrease brain oxidative stress and AChE, monoamine oxidase B, total nitric oxide synthase in senescent mice induced by D-galactose, and may be used to treat AD [65,66].

In the present study, the value for CTC from CsAE was 11.43 ± 0.041 mg TAE/g DW. In a survey of Othmen et al. [60], the condensed tannins content from *C. siliqua* leaf extracts reached 18.52 ± 0.03 g catechin equivalents (CE) 100/g DW. Highly polymerized condensed tannins containing a flavan nucleus were previously isolated from *C. siliqua* pods [67]. 

### 3.4. UHPLC Analysis

**Figure 2:**
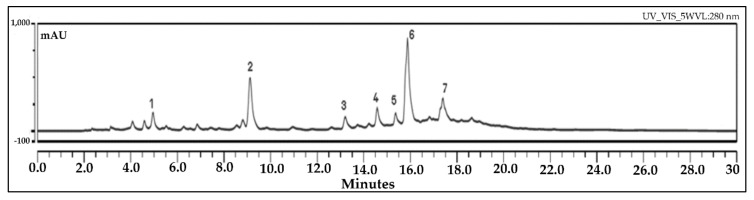
The ultra-high-performance liquid chromatography (UHPLC) chromatogram of the aqueous extract of *C. siliqua* (CsAE) indicating the picks following: 1. Gallic acid; 2. Epicatechin; 3. Chlorogenic acid; 4. Caffeic acid; 5. Quercetin-3-glucoside; 6. Luteolin-7-glucoside; 7. Apigenin-7-glucoside (numbers refer to Table 1).

The registered chromatogram is indicated in Figure 2 and shows the main peaks observed for the investigated *C. siliqua* sample at 280 nm, typical for flavonoids. The standard deviation values (expressed as %) varied between 0.06 and 0.18.

The major compound of the CsAE was luteolin-7-glucoside (23.78 w/w in the extract) followed by epicatechin (17.79 w/w in the extract), apigenin-7-glucoside (7.50 w/w in the extract), quercetin-3-glucoside (7.41 w/w in the extract), caffeic acid (4.84 w/w in the extract), gallic acid (4.54 w/w in the extract), and chlorogenic acid (3.59 w/w in the extract) (Table 1).

The results indicate that flavonoids amount up to three times more than the phenolic acids and twice as the catechin. Moreover, such quantities indicate that *C. siliqua* leaves represent a good source of natural bioactive compounds. No aglycons were identified in our sample. Goulas and Georgiou [68] demonstrated by HPLC analysis the presence of seven well-known phenolic antioxidants (gallic acid, syringic acid, catechin, epicatechin, quercetin, rutin, and myricetin) in the chemical composition of the carob extract. Among them, gallic acid was the main phenolic compound contained in the water extract. Moreover, significant amounts of rutin were noticed in the water extract, too. Corsi et al. [69] reported the presence of gallic acid, (−) epigallocatechin-3-gallate, and (−) epicatechin-3-gallate in the pod and leaf of *C. siliqua* extracts by HPLC analysis. 

### 3.5. Determination of Antioxidant Activity

The antioxidant activity of the *C. siliqua* aqueous extract is mainly linked to the phenolic compounds. Data from Table 2 reported the results of the antioxidant activities of CsAE using three methods: the capacity of scavenging DPPH radical, to reduce and to chelate iron. 

CsAE showed high anti-DPPH activity with IC_50_ = 0.116 ± 0.002 mg/mL, a value that is near to that of ascorbic acid used as a reference drug. CsAE exhibited a high FRAP with an EC_50_ = 0.123 ± 0.003 mg/mL, which is slightly inferior to the BHT used as standard. CsAE showed a low ability to chelate iron 0.971 ± 0.006 mg/mL, while the EDTA showed the highest chelating ability with an IC_50_ of 0.117 ± 0.0005 mg/mL. We can deduce that the iron-chelating activity of CsAE was low compared with ferric reducing power and DPPH scavenging assay. Our results are in line with other studies that reported the antioxidant activity of *C. siliqua* extracts. The DPPH^.^ is a free radical relatively stable [32,70] commonly used as an inexpensive, simple, and rapid test to estimate the capacity of extracts and active compounds to act as antioxidants by scavenging free radicals [71]. Othmen et al. [59] showed that both aqueous and ethanolic extracts of *C. siliqua* were able to trap free radicals. In contrast, the ethanolic extract presented the highest radical scavenging activity in terms of DPPH radicals, reaching 178.33 ± 2.12 g TE 100 g^−1^ DW. In another study, Goulas and Georgiou [66] describe the antioxidant activity of the *C. siliqua* extracts, as evidenced by the DPPH IC_50_ radical scavenging activity (1.9 ± 0.2 mg/mL) and FRAP data (339.7 ± 4.0 mg FeSO4 100 g^−1^). As reported by Custódio et al. [72], the methanolic extract of *Galhosa*, the female *C. siliqua* species from Spain, showed the highest DPPH scavenging power. According to the study carried out in Turkey, CsAE exhibited high DPPH inhibition percentage [58]. The research conducted on the ethyl acetate fraction in Tunisia revealed a tremendous antioxidant activity demonstrated by a low IC_50_ value [40]. Amessis-Ouchemoukh et al. [73] have reported that the hydroethanolic extract of *C. siliqua* pods presented a high DPPH inhibition. The aqueous extract from three varieties of Algerian *C. siliqua* in the unripe stage also showed a high anti-DPPH capacity [74]. The DPPH discoloration degree in a solution reveals the ability of extracts to release H^+^ protons [75], and it is likely due to the presence of products having the capacity to interact with free radicals as electron donors [76] and, therefore, inhibiting the ROS such the hydroxyl radical, superoxide anion [77]. A dose-dependent relationship was observed between the concentration of CsAE and its antioxidant activity. DPPH scavenging capacity of plant extracts increases when the level of the OH^-^ groupings present in the aromatic rings rise [78]. However, other non-phenolic compounds present in the extracts may have an antioxidant effect [79].

CsAE showed an excellent capacity to reduce iron, significantly near to that of BHT. This result is hugely following the study of Benchikh et al. [74], which have shown that aqueous extracts of three varieties ripe carob exhibited a high FRAP. As noted by Amessis-Ouchemoukh et al. [73], the hydroethanolic extract of carob pods presented a great FRAP. 

It was suggested that the chelation of transition metals presents one of the main strategies used to investigate the antioxidant activity of plant extracts [80]. The presence of chelation factors allows the decrease of the red color of the ferrozine-Fe^2+^ complex in solution [81,82]. Transition-metal ions play an essential role in the oxidation process via Fenton reaction [83]; iron overload leads to the formation of lipid peroxidation products, which are involved in the onset of several chronic diseases [84] especially neurodegenerative diseases [85]. Therefore, iron chelators could be used as neuroprotective compounds against PD [86]. In the current study, CsAE presented a moderate ability to chelate iron, which was significantly inferior to that of EDTA. 

According to the study of El Hajaji et al. [44], the methanolic extracts from a variety of Moroccan carob barks showed good antioxidant power *in vitro*. It was previously reported that three varieties of *C. siliqua* leaves from Morocco exhibited high antioxidant activity *in vitro* [27]; from our results, we can deduce that CsAE could be used as a potential source of antioxidants. However, among 30 Moroccan medicinal plants, the CsAE from pods has not shown good antioxidant activity *in vitro* [87]. The same result was reported in the study carried out on multitude types of *C. siliqua* pods extracts from Algeria [74]. This difference could be explained by the fact that the antioxidant power of phenolic compounds is due to their high redox potential, which allows interacting as hydrogen donors, reducers, and singlet oxygen (^1^O_2_) quenchers [71]. The structure of phenolic compounds is also involved in their antioxidant activity. Indeed, the presence of hydroxyls groupings in the aromatic rings of flavonoids at 3′, 4′, and 5′ position increases their antioxidant ability. In addition, phenolic compounds with two adjacent hydroxyl groupings can participate in the chelation process of transition metal ions such the iron and copper [88]. 

### 3.6. Acetylcholinesterase Inhibitory Assay

**Figure 3:**
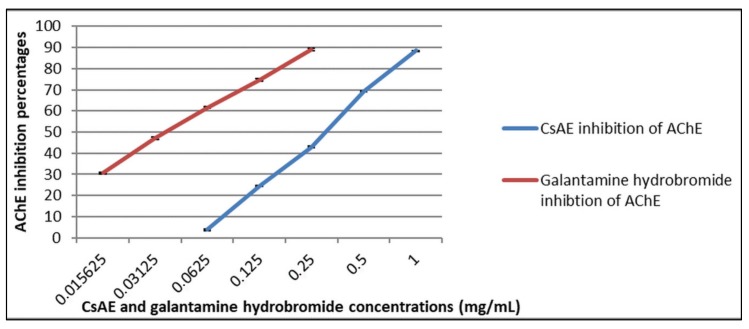
Inhibition percentages of acetylcholinesterase (AChE) exhibited by different concentrations of CsAE (0.0625–1 mg/mL) and galantamine hydrobromide (0.015–0.25 mg/mL). Coefficient and Regression equation of inhibition displayed by CsAE: *y* = 21.398*x* − 61.181, *R^2^* = 0.9972, IC_50_ = 0.29 ± 0.004 mg/mL. Coefficient and Regression equation of inhibition displayed by galantamine hydrobromide: *y* = 14.406*x* + 17.386, *R^2^* =0.9985, IC_50_ = 0.03 ± 0.0005 mg/mL.

As shown in Figure 3, CsAE exhibited anti-AChE activity in a dose-dependent manner. CsAE showed a high inhibitory activity against AChE with a maximal inhibition percentage of 88.53 ± 0.08 at 1 mg/mL of the extract and an IC_50_ value of 0.29 ± 0.004 mg/mL. The doses of 0.125 mg/mL (*p* < 0.0001) and 0.5 mg/mL (*p* < 0.001) of CsAE revealed a significant AChE inhibitory activity as compared to galantamine hydrobromide, a well-known AChE inhibitor, showing a maximal inhibition percentage of 98.05 ± 0.63 at 0.25 mg/mL and an IC_50_ value of 0.03 ± 0.0005 mg/mL. Our results agree with previous studies showing *in vitro* inhibitory activity on AChE of *C. siliqua* extract [34]. Moreover, our group recently demonstrated that the methanolic extract of *C. siliqua* possessed *in vitro* potential inhibition of AChE [89]. 

### 3.7. Effects on Anxiety-Like Behavior in NTT Test and on Spatial Memory in Y-Maze Test

**Figure 4:**
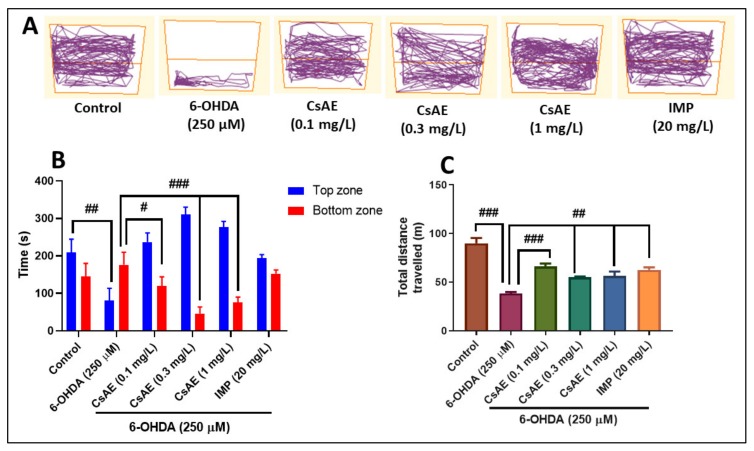
*Ceratonia siliqua* aqueous extract (CsAE, 0.1, 0.3, and 1 mg/L) ameliorated locomotion pattern and reduced anxiety in the NTT test. (**A**) Representative locomotion tracking pattern of the control, 6-hydroxydopamine (6-OHDA) (250 µM), CsAE (0.1, 0.3, and 1 mg/L), and imipramine (IMP) (20 mg/L) treated groups. (**B**) Represent the time spent in the top/bottom zone by zebrafish in the tank in different groups. (**C**) Represent the total distance travelled by zebrafish in the tank in different groups. Values are means ± S.E.M. (*n* = 10). For Tukey’s *post hoc* analyses: (**B**) Control vs. 6-OHDA (250 µM): ##*p* < 0.001, 6-OHDA (250 µM) vs. CsAE (0.1 mg/L): # *p* < 0.01, 6-OHDA (250 µM) vs. CsAE (0.3 mg/L): ### *p* < 0.0001 and 6-OHDA (250 µM) vs. CsAE (1 mg/L): ### *p* < 0.0001; (**C**) Control vs. 6-OHDA (250 µM): ### *p* < 0.0001, 6-OHDA (250 µM) vs. CsAE (0.1 mg/L): ###p < 0.0001, 6-OHDA (250 µM) vs. CsAE (0.3 mg/L): ## *p* < 0.001 and 6-OHDA (250 µM) vs. CsAE (1 mg/L): ## *p* < 0.001.

Figure 4 shows the results of 6-OHDA (250 µM) and CsAE (0.1, 0.3, and 1 mg/L) treatment of anxiety-like behavior within the NTT test. Representative locomotion tracking pattern (Figure 4A) highlighted the discrepancies between the top and bottom areas in swimming traces. It revealed that 6-OHDA-treated zebrafish exhibited a preference for the bottom zone, indicating an anxiogenic profile. Moreover, the 6-OHDA treatment increased the time spent in the bottom zone (*p* < 0.01), as well as decreased the time spent in the top zone (*p* < 0.001) as compared to the control group (Figure 4B). Reducing the time spent in the top zone of the tank suggests the anxiogenic-like profile of 6-OHDA. By decreasing the total distance traveled in the tank, 6-OHDA treatment created a hypolocomotor effect (Figure 4C) compared to control. By comparison, the time spent in the top zone of the tank (Figure 4B) indicates the anxiolytic-like result of CsAE, particularly at the dose of 0.3 mg/L. Besides, CsAE treatment avoids 6-OHDA anxiogenic effect as shown by decreasing of the time spent in the bottom zone of the tank (*p* < 0.01 for 0.1 mg/L and *p* < 0.0001 for 0.3 and 1 mg/L) (Figure 4B) and by increasing of the total distance travelled (*p* < 0.0001 for 0.1 mg/L and *p* < 0.001 for 0.3 and 1 mg/L) (Figure 4C) relative to 6-OHDA alone-treated zebrafish. Moreover, 6-OHDA alone-treated zebrafish exhibited a decreased total distance travelled in the tank as compared to the control group (*p* < 0.0001), suggesting anxiogenic effects) (Figure 4C). IMP (20 mg/L) used as the positive reference drug exhibited an anxiolytic profile, as evidenced by decreasing the time spent in the bottom zone of the tank and by increasing the total distance travelled in the 6-OHDA-treated zebrafish. 

**Figure 5:**
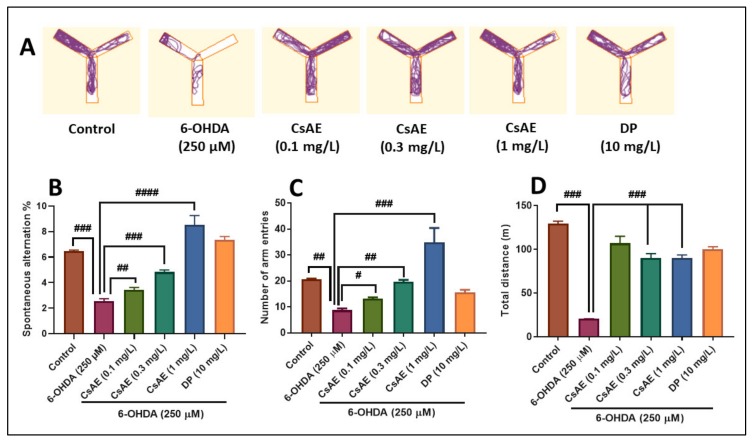
*Ceratonia siliqua* aqueous extract (CsAE, 0.1, 0.3, and 1 mg/L) improved locomotion pattern and memory in the Y-maze test. (**A**) Representative locomotion tracking of the control, 6-OHDA (250 µM) CsAE (0.1, 0.3, and 1 mg/L), and donepezil (DP) (10 mg/L) treated groups. (**B**) Represent the percentage of spontaneous alternation in the Y-maze in different groups. (**C**) Represent the number of arm entries in the Y-maze in different groups. (**D**) Represent the total distance travelled by zebrafish in the Y-maze in different groups. Values are means ± S.E.M. (*n* = 10). For Tukey’s *post hoc* analyses: (**B**) Control vs. 6-OHDA (250 μM): ###*p* < 0.0001, 6-OHDA (250 µM) vs. CsAE (0.1 mg/L): ## *p* < 0.001, 6-OHDA (250 µM) vs. CsAE (0.3 mg/L): ### *p* < 0.0001 and 6-OHDA (250 µM) vs. CsAE (1 mg/L): #### *p* < 0.00001; (**C**) Control vs. 6-OHDA (250 μM): ## *p* < 0.001, 6-OHDA (250 µM) vs. CsAE (0.1 mg/L): # *p* < 0.01, 6-OHDA (250 µM) vs. CsAE (0.3 mg/L): ## *p* < 0.001 and 6-OHDA (250 µM) vs. CsAE (1 mg/L): ### *p* < 0.0001; (**D**) Control vs. 6-OHDA (250 μM): ### *p* < 0.0001, 6-OHDA (250 µM) vs. CsAE (0.3 mg/L): ### *p* < 0.0001 and 6-OHDA (250 µM) vs. CsAE (1 mg/L): ### *p* < 0.0001.

Figure 5 illustrates the effects of 6-OHDA (250 µM) and CsAE (0.1, 0.3, and 1 mg/L) treatment on the Y-maze spatial memory. Representative locomotion tracking pattern (Figure 5A) illustrates the differences between the Y-maze arms in swimming traces and shows that 6-OHDA treated group traveled less distance in the Y-maze, suggesting hypolocomotion. Moreover, the administration of 6-OHDA induced memory deficits as evidenced by decreased the percentage of spontaneous alternation (*p* < 0.0001) (Figure 5B) as compared to the control group. Administration of CsAE significantly counters the 6-OHDA action induced-memory impairment, as evidenced by increased the percentage of spontaneous alternation in a dose-dependent manner. Reducing the percentage of spontaneous alternation suggests the memory impairment effect of 6-OHDA.

Furthermore, 6-OHDA administration affects locomotion, as illustrated by decreased number of arm entries (*p* < 0.001) (Figure 5C) and a reduced of the total distance (*p* < 0.0001) (Figure 5D) as compared to the control group. By contrast, the administration of CsAE in the 6-OHDA fish significantly improved locomotion, and increased total distance travelled in the Y-maze test. Our findings suggested that CsAE displayed anxiolytic and cognitive-enhancing effects, which could be attributed to the presence of the bioactive compounds shown in Table 1. Recent studies have identified many physiological effects of *C. siliqua* fruit and its by-products due to the existence of bioactive compounds that may be important to the promotion of human health and chronic disease prevention [68,90]. It has been demonstrated that *C. siliqua* is a source of bioactive ingredients with antioxidant, anti-hypertensive, and anti-inflammatory potential [91]. Our results are in line with those reported by Alzoubi et al. [92], who demonstrated that the methanolic extract from *C. siliqua* prevented short-term memory deficit induced by chronic stress in rats, probably as a result of avoiding reduction in the brain-derived neurotrophic factor (BDNF) levels in the hippocampus. Moreover, the CsAE exhibited an anxiolytic-like effect and prevented emotional behavior impairment and metabolic disorders induced by estrogen deficiency in rats [93]. 

### 3.8. Effects on the Brain AChE Activity

**Figure 6:**
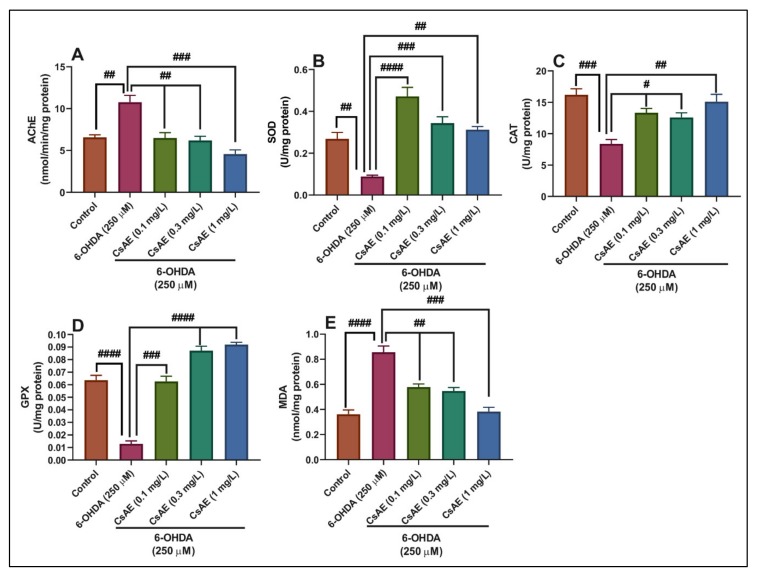
*Ceratonia siliqua* aqueous extract (CsAE, 0.1, 0.3, and 1 mg/L) exhibited an anti-AChE effect and improved brain antioxidant status. The enzyme’s specific activities: (**A**) Acetylcholinesterase (AChE), (**B**) Superoxide dismutase (SOD), (**C**) Catalase (CAT), and (**D**) Glutathione peroxidase (GPX) and (**E**) Malondialdehyde (MDA) level. Values are means ± S.E.M. (*n* = 10). For Tukey’s *post hoc* analyses: (**A**) Control vs. 6-OHDA (250 μM): ##*p* < 0.001, 6-OHDA (250 µM) vs. CsAE (0.1 mg/L): ## *p* < 0.001, 6-OHDA (250 µM) vs. CsAE (0.3 mg/L): ## *p* < 0.001 and 6-OHDA (250 µM) vs. CsAE (1 mg/L): #### *p* < 0.0001; (**B**) Control vs. 6-OHDA (250 μM): ## *p* < 0.001, 6-OHDA (250 µM) vs. CsAE (0.1 mg/L): #### *p* < 0.00001, 6-OHDA (250 µM) vs. CsAE (0.3 mg/L): ### *p* < 0.0001 and 6-OHDA (250 µM) vs. CsAE (1 mg/L): ## *p* < 0.001; (**C**) Control vs. 6-OHDA (250 μM): ### *p* < 0.0001, 6-OHDA (250 µM) vs. CsAE (0.1 mg/L): # *p* < 0.01, 6-OHDA (250 µM) vs. CsAE (0.3 mg/L): # *p* < 0.01 and 6-OHDA (250 µM) vs. CsAE (1 mg/L): ## *p* < 0.001; (**D**) Control vs. 6-OHDA (250 μM): #### *p* < 0.00001, 6-OHDA (250 µM) vs. CsAE (0.1 mg/L): ### *p* < 0.0001, 6-OHDA (250 µM) vs. CsAE (0.3 mg/L): ### *p* < 0.0001 and 6-OHDA (250 µM) vs. CsAE (1 mg/L): #### *p* < 0.00001; and (**E**) Control vs. 6-OHDA (250 μM): #### *p* < 0.00001, 6-OHDA (250 µM) vs. CsAE (0.1 mg/L): ## *p* < 0.001, 6-OHDA (250 µM) vs. CsAE (0.3 mg/L): ## *p* < 0.001 and 6-OHDA (250 µM) vs. CsAE (1 mg/L): ### *p* < 0.0001.

The effect of CsAE on AChE activity in the zebrafish brain was examined. The 6-OHDA-treated zebrafish showed significantly increased AChE activity in the brain of zebrafish as compared to the control (*p* < 0.001). By contrast, CsAE administration (0.1, 0.3, and 1 mg/L) significantly inhibited AChE in the brain by 6.49 ± 0.63 (*p* < 0.001), 6.18 ± 0.51 (*p* < 0.001), and 4.57 ± 0.49 (*p* < 0.0001) nmol/min/mg protein compared with the 6-OHDA-treated group (Figure 6A). 

AChE is an enzyme localized in the nervous system and muscles of vertebrates and humans [94]. The significant role of this enzyme is the termination of transmission at the cholinergic synapses [95] by hydrolyzing acetylcholine to choline and acetate [96]. The inhibition of AChE could be used to treat AD [97]. Moreover, the depletion of acetylcholine is also detected in PD, participating in dementia as a non-motor symptom of this pathology [98]. Interestingly, alterations in the cortical cholinergic pathways can affect cognitive capacities and lead to dementia in PD patients [99]. Several authors have shown that *C. siliqua*—mainly the leaves—exhibited an excellent ability *in vitro* to inhibit AChE [100]. However, no studies were found about inhibition *in vivo*. In contrast, Uysal et al. [58] showed that CsAE inhibited butyrylcholinesterase (BChE) but not inhibited AChE. 

### 3.9. Effects on the Brain SOD, CAT, and GPX Specific Activities, and the MDA Level

The 6-OHDA injection significantly decreased the SOD specific activity (*p* < 0.001) (Figure 6B) in the zebrafish brain as compared to the control group, suggesting facilitation of the oxidative stress. The administration of CsAE in three doses (0.1; 0.3, and 1 mg/L) significantly prevented the decreased SOD activity in the 6-OHDA-treated zebrafish, but 0.1 mg/L remains the best ameliorative dose of SOD (*p* < 0.00001) as compared to 6-OHDA alone-treated zebrafish. Our results also showed a significant decrease in CAT activity following the injection of 6-OHDA as compared to control (*p* < 0.0001) (Figure 6C). The administration of the three doses of CsAE (0.1; 0.3, and 1 mg/L) in the 6-OHDA zebrafish significantly prevented the decrease of the same antioxidant enzyme, especially the dosage of 1 mg/L; however, the levels were inferior to those of control. We also observed that the 6-OHDA injection produced marked decreases in the activity of GPX (*p* < 00001) (Figure 6D) and the three doses of CsAE significantly exhibited a high power to enhance the activity of GPX, especially the doses of 0.3 and 1 mg/L (*p* < 0.00001) in zebrafish brain compared with 6-OHDA-treated zebrafish. The treatment with the 6-OHDA increases the levels of MDA in the zebrafish brain as compared to control (*p* < 0.00001), and the administration of CsAE inhibits the MDA, especially with 1 mg/L (Figure 6E).

The brain is sensitive to oxidative stress, which activates the production of anion superoxide and hydrogen peroxide [101]. ROS generated leads to neurons loss and, consequently, cognitive impairment observed in PD [21]. The antioxidant enzymes such as SOD, CAT, and GPX play a vital role in the human body’s defense against oxidative stress [102]. The SOD is a metalloenzyme, ubiquitous [103], and having the capacity to scavenge the superoxide radical [104]; this enzyme catalyzes the dismutation of superoxide anion into molecular oxygen (O_2_) and hydrogen peroxide (H_2_O_2_) [105]. The GPX belongs to the family of isozymes using GSH to reduce H_2_O_2_ and lipid hydroperoxides [103]. CAT is the essential detoxification enzyme; it is indispensable for the direct dismutation of H_2_O_2_ into H_2_O and O_2_ [103]. MDA is the main marker of lipid peroxidation [102]; it is a reactive aldehyde resulting from the peroxidation of polyunsaturated fatty acids in the cells membrane [106].

The study of Akkaya and Yilmaz [107] pointed out that the methanolic extract of *C. siliqua* from Turkey has a good capacity to reduce MDA levels *in vitro*. Corsi et al. [69] suggested that the CsAE from Italy had a strong inhibition power of T1 cell proliferation. In addition, the CsAE from pods from Tunisia exerted a high antioxidant capacity in the kidney, liver and brain of adult male Wistar rats [108]. Rtibi et al. [109] found that the CsAE from pods exhibited *in vivo* antioxidant activity in ethanol-treated male swiss albino mice and male Wistar rats. Moreover, pretreatment of male albino Wistar rats with the ethyl acetate fraction from the *C. siliqua* leaves from Tunisia prevented oxidative damages induced by carbon tetrachloride (CCl_4_) in the kidney and liver [40]. Additionally, the ethanolic extract from *C. siliqua* leaves was found to be effective in protecting against oxidative damage induced by cisplatin in the kidney of albino male mice [110]. 

**Figure 7:**
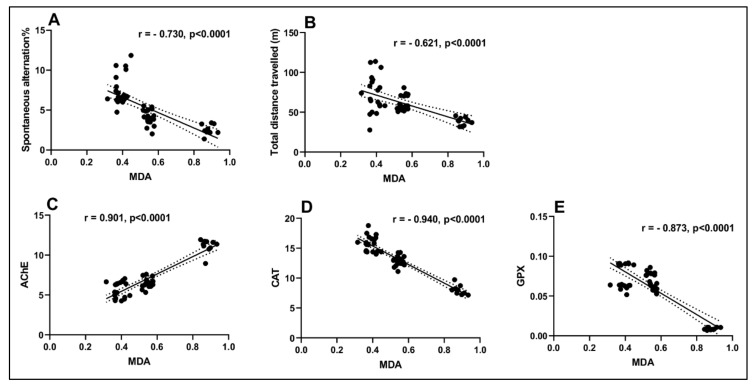
Correlation analyses between behavioral and biochemical parameters (Pearson’s correlation, *n* = 10): (**A**) spontaneous alternation% vs. MDA: *r* = −0.730, *p* < 0.0001; (**B**) total distance travelled vs. MDA: r = −0.621, *p* < 0.0001; (**C**) AChE vs. MDA: *r* = 0.901, *p* < 0.0001; (**D**) CAT vs. MDA: *r* = −0.940, *p* < 0.0001 and (**E**) GPX vs. MDA: *r* = −0.873, *p* < 0.0001. Data expressed are spontaneous alternation %, total distance travelled (m), AChE (nmol/min/mg protein), CAT (U/mg protein), GPX (U/mg protein), and MDA (nmol/mg protein).

Pearson correlation coefficient (*r*) was used to test the linear association between memory scores, antioxidant enzymes, and lipid peroxidation (Figure 7). Significant negative correlations between the spontaneous alternation percentage vs. MDA (*n* = 10, *r* = −0.730, *p* < 0.0001) (Figure 7A) and between the total distance travelled in the tank vs. MDA (*n* = 10, *r* = −0.621, *p* < 0.0001) (Figure 7B) were observed. The negative value of *r* indicates that the improvement of memory scores in specific tests such as Y-maze and NTT is well correlated with a decreased level of MDA, a marker of lipid peroxidation. Moreover, strong positive correlation was noticed by linear regression between AChE vs. MDA (*n* = 10, *r* = 0.901, *p* < 0.0001) (Figure 7C). However, significant negative correlations between CAT vs. MDA (*n* = 10, *r* = −0.940, *p* < 0.0001) (Figure 6D) and between GPX vs. MDA (*n* = 10, *r* = −0.873, *p* < 0.0001) (Figure 7E) were reported when linear regression was calculated. In this case, the positive and negative values of the *r* indicate that decreasing of AChE specific activity as well as increasing of CAT and GPX specific activities is well correlated with a low MDA level. Custodio et al. [100] reported a correlation between in vitro antioxidant activity and the inhibitory activity on α-amylase, α-glucosidase, AChE, and BChE following the administration of the *C. siliqua* extract. Using the *r* determination, we have shown that improving memory performance in 6-OHDA-treated zebrafish is linked to increased antioxidant enzyme activity along with a decreased AChE activity and the level of MDA (lipid peroxidation), which promotes the CsAE neuroprotective profile.

## 4. Conclusions

By using UHPLC analysis, we showed that the major compound of the CsAE was luteolin-7-glucoside, followed by epicatechin, apigenin-7-glucoside, quercetin-3-glucoside, caffeic acid, gallic acid, and chlorogenic acid. Furthermore, the obtained data accomplished the proposed objectives, showing that CsAE attenuated memory deficits and anxiety resulting from 6-OHDA treatment by a mechanism implying restoring of brain antioxidant status and regulation of AChE activity. Consequently, the present study indicated that CsAE could be regarded as an alternative source of bioactive compounds that could enhance memory processes in the 6-OHDA zebrafish model of PD. 

## Figures and Tables

**Table 1:** The most important compounds identified in *C. siliqua* aqueous extract by UHPLC analysis.

Sample	Compound (w/w in the Extract)						
	1	2	3	4	5	6	7
CsAE	4.54	17.79	3.59	4.84	7.41	23.78	7.50

1. Gallic acid; 2. Epicatechin; 3. Chlorogenic acid; 4. Caffeic acid; 5. Quercetin-3-glucoside; 6. Luteolin-7-glucoside; 7. Apigenin-7-glucoside.

**Table 2:** Antioxidant activities of the *C. siliqua* aqueous extract and standards.

Antioxidant Activity Assays	IC_50_ (mg/mL)		Coefficient and Regression Equation		Maximal Inhibition Percentages (%)	
1,1-Diphenyl-2-picrylhydrazyl **(**DPPH) scavenging power	CsAE	Ascorbic acid	CsAE	Ascorbic acid	CsAE	Ascorbic acid (1 mg/mL)
	0.116 ± 0.002	0.106 ± 0.0005	*y* = −10.929*x* + 97.105 *R^2^* = 0.9948	*y* = 15.48*x*−12.535 *R^2^* = 0.9915	84.65 ± 0.01	92.14 ± 0.04
Ferric reducing antioxidant power **(**FRAP)	CsAE (EC_50_)	BHT (EC_50_)	CsAE	BHT	CsAE	BHT
	0.123 ± 0.003	0.118 ± 0.00	*y* = −0.101*x* + 0.915 *R^2^* = 0.9999	*y* = 0.311*x* − 0.017 *R^2^* = 0.9958	-	-
Iron chelating activity	CsAE	EDTA	CsAE	EDTA	CsAE	EDTA (1 mg/mL)
	0.971 ± 0.006	0.117 ± 0.0005	*y* = −12.356*x* + 62.71 *R^2^* = 0.9939	*y* = 15.927*x* − 12.002 *R^2^* = 0.9914	51.5 ± 0.02	94.96 ± 0.003

The half maximal inhibitory concentration (IC_50_) and the half maximal effective concentration (EC_50_) of the *C. siliqua* aqueous extract (CsAE) were compared with those of the standards: There is no significant difference between CsAE and standards. Butylated hydroxytoluene (BHT), Ethylenediaminetetraacetic acid (EDTA).

## References

[1] Lee E., Park H.R., Ji S.T., Lee Y., Lee J. (2013). Baicalein attenuates astroglial activation in the 1-methyl-4-phenyl-1,2,3,4-tetrahydropyridine-induced Parkinson’s disease model by downregulating the activations of nuclear factor-κB, ERK, and JNK. J. Neurosci. Res..

[2] Daviaud N., Garbayo E., Lautram N., Franconi F., Lemaire L., Perez-Pinzon M., Montero-Menei C.N. (2013). Modeling nigrostriatal degeneration in organotypic cultures, a new ex vivo model of Parkinson’s disease. Neuroscience.

[3] Gaki G.S., Papavassiliou A.G. (2014). Oxidative Stress-Induced Signaling Pathways Implicated in the Pathogenesis of Parkinson’s Disease. NeuroMolecular Med..

[4] Pradhan S.S., Salinas K., Garduno A.C., Johansson J.U., Wang Q., Manning-Bog A., Andreasson K.I. (2016). Anti-Inflammatory and Neuroprotective Effects of PGE2 EP4 Signaling in Models of Parkinson’s Disease. J. Neuroimm. Pharmacol..

[5] Muñoz-Manchado A.B., Villadiego J., Suárez-Luna N., Bermejo-Navas A., Garrido-Gil P., Labandeira-Garcia J.L., Echevarría M., López-Barneo J., Toledo-Aral J.J., Echevarría M. (2013). Neuroprotective and reparative effects of carotid body grafts in a chronic MPTP model of Parkinson’s disease. Neurobiol. Aging.

[6] Prediger R.D., Matheus F.C., Schwarzbold M.L., Lima M.M.S., Vital M.A. (2012). Anxiety in Parkinson’s disease: A critical review of experimental and clinical studies. Neuropharmacology.

[7] Kehagia A.A., Barker R.A., Robbins T.W. (2010). Neuropsychological and clinical heterogeneity of cognitive impairment and dementia in patients with Parkinson’s disease. Lancet Neurol..

[8] Erro R., Santangelo G., Picillo M., Vitale C., Amboni M., Longo K., Costagliola A., Pellecchia M.T., Allocca R., Rosa A. (2012). Link between non-motor symptoms and cognitive dysfunctions in de novo, drug-naive PD patients. J. Neurol..

[9] Gallagher D.A., Schrag A. (2012). Psychosis, apathy, depression and anxiety in Parkinson’s disease. Neurobiol. Dis..

[10] Sekeroglu N., Deniz F.S.S., Orhan I.E., Gulpinar A.R., Kartal M., Sener B., Şekeroǧlu N. (2012). In vitro prospective effects of various traditional herbal coffees consumed in Anatolia linked to neurodegeneration. Food Res. Int..

[11] Khan M.M., Ahmad A., Ishrat T., Khan M.B., Hoda N., Khuwaja G., Raza S.S., Khan A., Javed H., Vaibhav K. (2010). Resveratrol attenuates 6-hydroxydopamine-induced oxidative damage and dopamine depletion in rat model of Parkinson’s disease. Brain Res..

[12] Wang M., Zhang Z., Cheang L.C.-V., Lin Z., Lee S.M.-Y. (2011). Eriocaulon buergerianum extract protects PC12 cells and neurons in zebrafish against 6-hydroxydopamine-induced damage. Chin. Med..

[13] Zhang C., Li C., Chen S., Li Z., Jia X., Wang K., Bao J., Liang Y., Wang X., Chen M. (2017). Berberine protects against 6-OHDA-induced neurotoxicity in PC12 cells and zebrafish through hormetic mechanisms involving PI3K/AKT/Bcl-2 and Nrf2/HO-1 pathways. Redox Biol..

[14] Panula P., Chen Y.-C., Priyadarshini M., Kudo H., Semenova S., Sundvik M., Sallinen V. (2010). The comparative neuroanatomy and neurochemistry of zebrafish CNS systems of relevance to human neuropsychiatric diseases. Neurobiol. Dis..

[15] Newman M., Ebrahimie E., Lardelli M. (2014). Using the zebrafish model for Alzheimer’s disease research. Front. Genet..

[16] Richetti S., Blank M., Capiotti K., Piato A., Bogo M., Vianna M.R.M., Bonan C.D. (2011). Quercetin and rutin prevent scopolamine-induced memory impairment in zebrafish. Behav. Brain Res..

[17] Santana S., Rico E.P., Burgos J. (2012). Can zebrafish be used as animal model to study Alzheimer’s disease?. Am. J. Neurodegener. Dis..

[18] Gulcin I., Bursal E., Şehitoğlu M.H., Bílsel M., Goren A.C. (2010). Polyphenol contents and antioxidant activity of lyophilized aqueous extract of propolis from Erzurum, Turkey. Food Chem. Toxicol..

[19] Bhat A.H., Dar K.B., Anees S., Zargar M.A., Masood A., Sofi M.A., Ganie S.A. (2015). Oxidative stress, mitochondrial dysfunction and neurodegenerative diseases; a mechanistic insight. Biomed. Pharmacother..

[20] Limón-Pacheco J., Gonsebatt M.E. (2009). The role of antioxidants and antioxidant-related enzymes in protective responses to environmentally induced oxidative stress. Mutat. Res. Toxicol. Environ. Mutagen..

[21] Kamdem J.P., Olalekan E.O., Hassan W., Kade I.J., Yetunde O., Boligon A., Athayde M.L., Souza D.O., Rocha J.B.T. (2013). Trichilia catigua (Catuaba) bark extract exerts neuroprotection against oxidative stress induced by different neurotoxic agents in rat hippocampal slices. Ind. Crop. Prod..

[22] Roberts R.A., Smith R.A., Safe S., Szabó C., Tjalkens R.B., Robertson F.M. (2010). Toxicological and pathophysiological roles of reactive oxygen and nitrogen species. Toxicology.

[23] Chong C.-M., Zhou Z.-Y., Razmovski-Naumovski V., Cui G.-Z., Zhang L.-Q., Sa F., Hoi P.-M., Chan K., Lee S.M.-Y. (2013). Danshensu protects against 6-hydroxydopamine-induced damage of PC12 cells in vitro and dopaminergic neurons in zebrafish. Neurosci. Lett..

[24] Giordano S., Darley-Usmar V., Zhang J. (2013). Autophagy as an essential cellular antioxidant pathway in neurodegenerative disease. Redox Biol..

[25] Petti S., Scully C. (2009). Polyphenols, oral health and disease: A review. J. Dent..

[26] Almanasrah M., Roseiro L.B., Lukasik R., Carvalheiro F., Brazinha C., Crespo J.G., Kallioinen M., Mänttäri M., Duarte L.C., Roseiro M.L.D.B.W. (2015). Selective recovery of phenolic compounds and carbohydrates from carob kibbles using water-based extraction. Ind. Crop. Prod..

[27] El Hajaji H., Lachkar N., Alaoui K., Cherrah Y., Farah A., Ennabili A., El Bali B., Lachkar M. (2010). Antioxidant properties and total phenolic content of three varieties of carob tree leaves from Morocco. Rec. Nat. Prod..

[28] Roseiro L.B., Duarte L.C., Oliveira D., Roque R., Bernardo-Gil M.G., Martins A., Sepúlveda C., Almeida J., Meireles M., Gírio F.M. (2013). Supercritical, ultrasound and conventional extracts from carob (Ceratonia siliqua L.) biomass: Effect on the phenolic profile and antiproliferative activity. Ind. Crop. Prod..

[29] Kasrati A., Jamali C.A., Fadli M., Bekkouche K., Hassani L., Wohlmuth H., Leach D., Abbad A. (2014). Antioxidative activity and synergistic effect of Thymus saturejoides Coss. essential oils with cefixime against selected food-borne bacteria. Ind. Crop. Prod..

[30] Oniszczuk A., Podgórski R. (2015). Influence of different extraction methods on the quantification of selected flavonoids and phenolic acids from Tilia cordata inflorescence. Ind. Crop. Prod..

[31] Kukula-Koch W., Aligiannis N., Halabalaki M., Skaltsounis A.-L., Glowniak K., Kalpoutzakis E. (2013). Influence of extraction procedures on phenolic content and antioxidant activity of Cretan barberry herb. Food Chem..

[32] Park E.-J., Jhon D.-Y. (2010). The antioxidant, angiotensin converting enzyme inhibition activity, and phenolic compounds of bamboo shoot extracts. LWT.

[33] Chonpathompikunlert P., Wattanathorn J., Muchimapura S. (2010). Piperine, the main alkaloid of Thai black pepper, protects against neurodegeneration and cognitive impairment in animal model of cognitive deficit like condition of Alzheimer’s disease. Food Chem. Toxicol..

[34] Custódio L., Escapa A.L., Fernandes E., Fajardo A., Aligué R., Alberício F., Neng N., Nogueira J.M.F., Romano A. (2011). In vitro cytotoxic effects and apoptosis induction by a methanol leaf extract of carob tree (*Ceratonia siliqua* L.). J. Med. Plants Res..

[35] Obeidat B., Alrababah M., Alhamad M., Gharaibeh M., Abu Ishmais M. (2012). Effects of feeding carob pods (*Ceratonia siliqua* L.) on nursing performance of Awassi ewes and their lambs. Small Rumin. Res..

[36] Tetik N., Yuksel E. (2014). Ultrasound-assisted extraction of d-pinitol from carob pods using Response Surface Methodology. Ultrason. Sonochem..

[37] Osório J., Osório J., Gonçalves S., David M.M., Correia M.J., Romano A. (2012). Carob trees (*Ceratonia siliqua* L.) regenerated in vitro can acclimatize successfully to match the field performance of seed-derived plants. Trees.

[38] Vekiari S.A., Ouzounidou G., Öztürk M., Görk G. (2011). Variation of quality characteristics in Greek and Turkish carob pods during fruit development. Procedia Soc. Behav. Sci..

[39] ElBatal H., Hasib A., Ouatmane A., Dehbi F., Jaouad A., Boulli A. (2016). Sugar composition and yield of syrup production from the pulp of Moroccan carob pods (Ceratonia siliqua L.). Arab. J. Chem..

[40] Hsouna A.B., Saoudi M., Trigui M., Jamoussi K., Boudawara T., Jaoua S., Feki A. (2011). El Characterization of bioactive compounds and ameliorative effects of Ceratonia siliqua leaf extract against CCl4 induced hepatic oxidative damage and renal failure in rats. Food Chem. Toxicol..

[41] Papagiannopoulos M., Wollseifen H.R., Mellenthin A., Haber B., Galensa R. (2004). Identification and Quantification of Polyphenols in Carob Fruits (*Ceratonia siliqua* L.) and Derived Products by HPLC-UV-ESI/MS n. J. Agric. Food Chem..

[42] Youssef M.K.E., El-Manfaloty M.M., Ali H.M. (2013). Assessment of proximate chemical composition, nutritional status, fatty acid composition and phenolic compounds of carob (*Ceratonia siliqua* L.). Food Public Heal..

[43] Sidina M.M., El Hansali M., Wahid N., Ouatmane A., Boulli A., Haddioui A. (2009). Fruit and seed diversity of domesticated carob (*Ceratonia siliqua* L.) in Morocco. Sci. Hortic..

[44] El Hajaji H., Lachkar N., Alaoui K., Cherrah Y., Farah A., Ennabili A., El Bali B., Lachkar M. (2011). Antioxidant activity, phytochemical screening, and total phenolic content of extracts from three genders of carob tree barks growing in Morocco. Arab. J. Chem..

[45] Chen G.-L., Chen S.-G., Xie Y.-Q., Chen F., Zhao Y.-Y., Luo C.-X., Gao Y.-Q. (2015). Total phenolic, flavonoid and antioxidant activity of 23 edible flowers subjected to in vitro digestion. J. Funct. Foods.

[46] Li H.-B., Wong C.-C., Cheng K.-W., Chen F. (2008). Antioxidant properties in vitro and total phenolic contents in methanol extracts from medicinal plants. LWT.

[47] Kusirisin W., Srichairatanakool S., Lerttrakarnnon P., Lailerd N., Suttajit M., Jaikang C., Chaiyasut C. (2009). Antioxidative activity, polyphenolic content and anti-glycation effect of some Thai medicinal plants traditionally used in diabetic patients. Med. Chem..

[48] Velázquez M.E., Tournier H., De Buschiazzo P.M., Saavedra G., Schinella G. (2003). Antioxidant activity of Paraguayan plant extracts. Fitoterapia.

[49] Chahmi N., Anissi J., Jennan S., Farah A., Sendide K., Hassouni M. (2015). El Antioxidant activities and total phenol content of Inula viscosa extracts selected from three regions of Morocco. Asian Pac. J. Trop. Biomed..

[50] Meghashri S., Kumar H.V., Gopal S. (2010). Antioxidant properties of a novel flavonoid from leaves of Leucas aspera. Food Chem..

[51] Chaiyana W., Okonogi S. (2012). Inhibition of cholinesterase by essential oil from food plant. Phytomedicine.

[52] Zhang J.-L., Souders C.L., Denslow N.D., Martyniuk C.J. (2017). Quercetin, a natural product supplement, impairs mitochondrial bioenergetics and locomotor behavior in larval zebrafish (Danio rerio). Toxicol. Appl. Pharmacol..

[53] Feng C.-W., Wen Z.-H., Huang S.-Y., Hung H.-C., Chen C.-H., Yang S.-N., Chen N.-F., Wang H.-M., Hsiao C.-D., Chen W.-F. (2014). Effects of 6-Hydroxydopamine Exposure on Motor Activity and Biochemical Expression in Zebrafish (Danio Rerio) Larvae. Zebrafish.

[54] Cachat J., Canavello P.R., Elkhayat S.I., Bartels B.K., Hart P., Elegante M.F., Beeson E.C., Laffoon A.L., Haymore W.A., Tien D.H. (2010). Video-Aided Analysis of Zebrafish Locomotion and Anxiety-Related Behavioral Responses. Viral Vector Approaches Neurobiol. Brain Dis..

[55] Dumitru G., El-Nashar H.A., Mostafa N.M., Eldahshan O.A., Boiangiu R.S., Todirascu-Ciornea E., Hritcu L., Singab A.N.B. (2019). Agathisflavone isolated from Schinus polygamus (Cav.) Cabrera leaves prevents scopolamine-induced memory impairment and brain oxidative stress in zebrafish (Danio rerio). Phytomedicine.

[56] Batista F.L.A., Lima L.M., Abrante I.A., De Araújo J.I.F., Batista F.L.A., Abrante I.A., Magalhães F.E.A., De Lima D.R., Lima M.D.C.L., Prado B.S.D. (2018). Antinociceptive activity of ethanolic extract of Azadirachta indica A. Juss (Neem, Meliaceae) fruit through opioid, glutamatergic and acid-sensitive ion pathways in adult zebrafish (Danio rerio). Biomed. Pharmacother..

[57] Smith P., Krohn R., Hermanson G., Mallia A., Gartner F., Provenzano M., Fujimoto E., Goeke N., Olson B., Klenk D. (1985). Measurement of protein using bicinchoninic acid. Anal. Biochem..

[58] Uysal S., Acqua S.D., Aktumsek A., Karatas S. (2016). Chemical and biological approaches on nine fruit tree leaves collected from the Mediterranean region of Turkey. J. Funct. Foods.

[59] Alali F., Tawaha K., El-Elimat T., Syouf M., El-Fayad M., Abulaila K., Nielsen S.J., Wheaton W.D., Iii J.O.F., Oberlies N.H. (2007). Antioxidant activity and total phenolic content of aqueous and methanolic extracts of Jordanian plants: An ICBG project. Nat. Prod. Res..

[60] Ben Othmen K., Elfalleh W., Beltrán J.M.G., Esteban M. (2020). Ángeles; Haddad, M. An in vitro study of the effect of carob (Ceratonia siliqua L.) leaf extracts on gilthead seabream (*Sparus aurata* L.) leucocyte activities. Antioxidant, cytotoxic and bactericidal properties. Fish Shellfish Immunol..

[61] Aboura I., Nani A., Belarbi M., Murtaza B., Fluckiger A., Dumont A., Benammar C., Tounsi M., Ghiringhelli F., Rialland M. (2017). Protective effects of polyphenol-rich infusions from carob (Ceratonia siliqua) leaves and cladodes of Opuntia ficus-indica against inflammation associated with diet-induced obesity and DSS-induced colitis in Swiss mice. Biomed. Pharmacother..

[62] Orhan I., Daglia M., Nabavi S., Loizzo M., Sobarzo-Sánchez E., Nabavi S. (2015). Flavonoids and dementia: An update. Curr. Med. Chem..

[63] Sassi A., Bouhlel I., Mustapha N., Mokdad-Bzeouich I., Chaabane F., Ghedira K., Chekir-Ghedira L. (2016). Assessment in vitro of the genotoxicity, antigenotoxicity and antioxidant of Ceratonia siliqua L. extracts in murine leukaemia cells L1210 by comet assay. Regul. Toxicol. Pharmacol..

[64] Vaya J., Mahmood S. (2006). Flavonoid content in leaf extracts of the fig (*Ficus carica* L.), carob (*Ceratonia siliqua* L.) and pistachio (*Pistacia lentiscus* L.). BioFactors.

[65] Gong Y.-S., Guo J., Hu K., Gao Y.-Q., Xie B.-J., Sun Z., Yang E.-N., Hou F.-L. (2016). Ameliorative effect of lotus seedpod proanthocyanidins on cognitive impairment and brain aging induced by d-galactose. Exp. Gerontol..

[66] Rusu M.E., Fizeșan I., Pop A., Gheldiu A.-M., Mocan A., Crișan G., Vlase L., Loghin F., Popa D.S., Tomuță I. (2019). Enhanced Recovery of Antioxidant Compounds from Hazelnut (*Corylus avellana* L.) Involucre Based on Extraction Optimization: Phytochemical Profile and Biological Activities. Antioxidants.

[67] Eldahshan O.A. (2011). Eldahshan Isolation and Structure Elucidation of Phenolic Compounds of Carob Leaves Grown in Egypt. Curr. Res. J. Biol. Sci..

[68] Goulas V., Georgiou E. (2019). Utilization of Carob Fruit as Sources of Phenolic Compounds with Antioxidant Potential: Extraction Optimization and Application in Food Models. Foods.

[69] Corsi L., Avallone R., Cosenza F., Farina F., Baraldi C., Baraldi M. (2002). Antiproliferative effects of Ceratonia siliqua L. on mouse hepatocellular carcinoma cell line. Fitoterapia.

[70] Končić M.Z., Kremer D., Karlovic K., Kosalec I. (2010). Evaluation of antioxidant activities and phenolic content of *Berberis vulgaris* L. and *Berberis croatica* Horvat. Food Chem. Toxicol..

[71] Loizzo M., Said A., Tundis R., Hawas U., Rashed K., Menichini F., Frega N.G., Menichini F. (2009). Antioxidant and Antiproliferative Activity of Diospyros lotus L. Extract and Isolated Compounds. Plant Foods Hum. Nutr..

[72] Custódio L., Fernandes E., Escapa A.L., López-Avilés S., Fajardo A., Aligué R., Albericio F., Romano A. (2009). Antioxidant activity andin vitroinhibition of tumor cell growth by leaf extracts from the carob tree (*Ceratonia siliqua*). Pharm. Biol..

[73] Amessis-Ouchemoukh N., Ouchemoukh S., Meziant N., Idiri Y., Hernanz D., Stinco C.M., Rodríguez-Pulido F.J., Heredia F.J., Madani K., Luis J. (2017). Bioactive metabolites involved in the antioxidant, anticancer and anticalpain activities of *Ficus carica* L., *Ceratonia siliqua* L. and *Quercus ilex* L. extracts. Ind. Crop. Prod..

[74] Benchikh Y., Louaileche H. (2014). Effects of extraction conditions on the recovery of phenolic compounds and in vitro antioxidant activity of carob (*Ceratonia siliqua* L.) pulp. Acta Bot. Gallica.

[75] Mbaebie B., Edeoga H., Afolayan A.J. (2012). Phytochemical analysis and antioxidants activities of aqueous stem bark extract of Schotia latifolia Jacq. Asian Pac. J. Trop. Biomed..

[76] Skotti E., Anastasaki E., Kanellou G., Polissiou M., Tarantilis P. (2014). Total phenolic content, antioxidant activity and toxicity of aqueous extracts from selected Greek medicinal and aromatic plants. Ind. Crop. Prod..

[77] Tagne R.S., Telefo P., Nyemb J.N., Yemele D.M., Njina S.N., Goka S.M.C., Lienou L.L., Kamdje A.H.N., Moundipa P.F., Farooq A.D. (2014). Anticancer and antioxidant activities of methanol extracts and fractions of some Cameroonian medicinal plants. Asian Pac. J. Trop. Med..

[78] Shahwar D., Raza M.A. (2012). Antioxidant potential of phenolic extracts of Mimusops elengi. Asian Pac. J. Trop. Biomed..

[79] Gonçalves S., Gomes D., Costa P., Romano A. (2013). The phenolic content and antioxidant activity of infusions from Mediterranean medicinal plants. Ind. Crop. Prod..

[80] Mathew S., Abraham T.E. (2006). In vitro antioxidant activity and scavenging effects of Cinnamomum verum leaf extract assayed by different methodologies. Food Chem. Toxicol..

[81] Kumaran D., Udayabanu M., Kumar M., Aneja R., Katyal A. (2008). Involvement of angiotensin converting enzyme in cerebral hypoperfusion induced anterograde memory impairment and cholinergic dysfunction in rats. Neuroscience.

[82] Terpinc P., Ceh B., Ulrih N.P., Abramovič H. (2012). Studies of the correlation between antioxidant properties and the total phenolic content of different oil cake extracts. Ind. Crop. Prod..

[83] Hinneburg I., Dorman H.D., Hiltunen R. (2006). Antioxidant activities of extracts from selected culinary herbs and spices. Food Chem..

[84] Sabir S.M., Rocha J., Rocha J.B.T. (2008). Water-extractable phytochemicals from Phyllanthus niruri exhibit distinct in vitro antioxidant and in vivo hepatoprotective activity against paracetamol-induced liver damage in mice. Food Chem..

[85] Zhang J., Zhang Y., Wang J., Cai P., Luo C., Qian Z., Dai Y., Feng H. (2010). Characterizing iron deposition in Parkinson’s disease using susceptibility-weighted imaging: An in vivo MR study. Brain Res..

[86] Zheng H., Gal S., Weiner L.M., Bar-Am O., Warshawsky A., Fridkin M., Youdim M.B. (2005). Novel multifunctional neuroprotective iron chelator-monoamine oxidase inhibitor drugs for neurodegenerative diseases: In vitro studies on antioxidant activity, prevention of lipid peroxide formation and monoamine oxidase inhibition. J. Neurochem..

[87] Dudonné S., Vitrac X., Coutière P., Woillez M., Mérillon J.-M. (2009). Comparative Study of Antioxidant Properties and Total Phenolic Content of 30 Plant Extracts of Industrial Interest Using DPPH, ABTS, FRAP, SOD, and ORAC Assays. J. Agric. Food Chem..

[88] Miguel M.G., Nunes S., Dandlen S.A., Cavaco A.M., Antunes M.D. (2010). Phenols and antioxidant activity of hydro-alcoholic extracts of propolis from Algarve, South of Portugal. Food Chem. Toxicol..

[89] Abidar S., Yildiz O., Degirmenci A., Amakran A., El Maadoudi M., Nhiri M. (2019). Glucose-mediated protein glycation: Contribution of methanolic extract of Ceratonia siliqua L. in protection and in vitro potential inhibition of acetylcholinesterase. J. Food Biochem..

[90] Benković M., Belščak-Cvitanović A., Bauman I., Komes D., Srečec S. (2017). Flow properties and chemical composition of carob ( Ceratonia siliqua L.) flours as related to particle size and seed presence. Food Res. Int..

[91] Rico D., Martín-Diana A.B., Martínez-Villaluenga C., Aguirre L., Silvan J.M., Dueñas M., De Luis D., Lasa A. (2019). In vitro approach for evaluation of carob by-products as source bioactive ingredients with potential to attenuate metabolic syndrome (MetS). Heliyon.

[92] Alzoubi K.H., Alibbini S., Khabour O.F., El-Elimat T., Al-Zubi M., Alali F. (2018). Carob (Ceratonia siliqua L.) Prevents Short-Term Memory Deficit Induced by Chronic Stress in Rats. J. Mol. Neurosci..

[93] Ammari M., Othman H., Rtibi K., Sakly M., Abdelmelek H. (2020). The Effects of Carob (*Ceratonia siliqua* L.) on Emotional Behavior Impairment and Metabolic Disorders Induced by Estrogen Deficiency in Rats. J. Med. Food.

[94] Scherer E.B., Da Cunha M.J., Matté C., Schmitz F., Netto C.A., Wyse A.T.S. (2010). Methylphenidate affects memory, brain-derived neurotrophic factor immunocontent and brain acetylcholinesterase activity in the rat. Neurobiol. Learn. Mem..

[95] Pegan K., Matkovic U., Mars T., Mis K., Pirkmajer S., Brecelj J., Grubic Z. (2010). Acetylcholinesterase is involved in apoptosis in the precursors of human muscle regeneration. Chem. Interactions.

[96] López M.D., Pascual-Villalobos M.J. (2010). Mode of inhibition of acetylcholinesterase by monoterpenoids and implications for pest control. Ind. Crop. Prod..

[97] Ingkaninan K., Temkitthawon P., Chuenchom K., Yuyaem T., Thongnoi W. (2003). Screening for acetylcholinesterase inhibitory activity in plants used in Thai traditional rejuvenating and neurotonic remedies. J. Ethnopharmacol..

[98] Bohnen N.I., Albin R.L. (2010). The cholinergic system and Parkinson disease. Behav. Brain Res..

[99] Chou K.L., Lenhart A., Koeppe R.A., Bohnen N.I. (2014). Abnormal MoCA and normal range MMSE scores in Parkinson disease without dementia: Cognitive and neurochemical correlates. Park. Relat. Disord..

[100] Custódio L., Patarra J., Albericio F., Neng N., Nogueira J., Romano A. (2015). In vitro antioxidant and inhibitory activity of water decoctions of carob tree (*Ceratonia siliqua* L.) on cholinesterases, α-amylase and α-glucosidase. Nat. Prod. Res..

[101] Syed F., Awasthi K.K., Chandravanshi L.P., Verma R., Rajawat N.K., Khanna V.K., John P.J., Soni I. (2018). Bifenthrin-induced neurotoxicity in rats: Involvement of oxidative stress. Toxicol. Res..

[102] Nabavi S.M., Nabavi S.F., Eslami S., Moghaddam A.H. (2012). In vivo protective effects of quercetin against sodium fluoride-induced oxidative stress in the hepatic tissue. Food Chem..

[103] Gill S., Tuteja N. (2010). Reactive oxygen species and antioxidant machinery in abiotic stress tolerance in crop plants. Plant Physiol. Biochem..

[104] Hybertson B.M., Gao B., Bose S.K., Mccord J.M. (2011). Oxidative stress in health and disease: The therapeutic potential of Nrf2 activation. Mol. Asp. Med..

[105] Pisoschi A.M., Pop A. (2015). The role of antioxidants in the chemistry of oxidative stress: A review. Eur. J. Med. Chem..

[106] You Y., Yoo S., Yoon H.-G., Park J., Lee Y.-H., Kim S., Oh K.-T., Lee J., Cho H.-Y., Jun W. (2010). In vitro and in vivo hepatoprotective effects of the aqueous extract from *Taraxacum officinale* (dandelion) root against alcohol-induced oxidative stress. Food Chem. Toxicol..

[107] Akkaya H., Yilmaz O. (2012). Antioxidant Capacity and Radical Scavenging Activity of Silybum marianum and Ceratonia siliqua. Ekoloji.

[108] Sebai H., Souli A., Chehimi L., Rtibi K., Amri M., El-Benna J., Sakly M. (2013). In vitro and in vivo antioxidant properties of Tunisian carob (*Ceratonia siliqua* L.). J. Med. Plants Res..

[109] Rtibi K., Jabri M.A., Selmi S., Souli A., Sebai H., El-Benna J., Amri M., Marzouki L. (2015). Gastroprotective effect of carob (Ceratonia siliqua L.) against ethanol-induced oxidative stress in rat. BMC Complement. Altern. Med..

[110] Ahmed M. (2010). Biochemical studies on nephroprotective effect of carob (*Ceratonia siliqua* L.) growing in Egypt. Nat. Sci..

