# Erratum: Abidar, S.; et al. The Aqueous Extract from *Ceratonia siliqua* Leaves Protects against 6-Hydroxydopamine in Zebrafish: Understanding the Underlying Mechanism. *Antioxidants* 2020, *9*, 304

**DOI:** 10.3390/antiox9060508

**Published:** 2020-06-09

**Authors:** Sara Abidar, Razvan Stefan Boiangiu, Gabriela Dumitru, Elena Todirascu-Ciornea, Amina Amakran, Oana Cioanca, Lucian Hritcu, Mohamed Nhiri

**Affiliations:** 1 Laboratoire de Biochimie et Génétique Moléculaire, Faculté des Sciences et Techniques, Université Abdelmalek Essaadi, Tanger Principal BP 416, Morocco; sara.abidar91@gmail.com (S.A.); amakran_amina@hotmail.com (A.A.); med.nhiri@gmail.com (M.N.); 2 Department of Biology, Faculty of Biology, Alexandru Ioan Cuza University of Iasi, 700506 Iasi, Romania; boiangiu.razvan@yahoo.com (R.S.B.); ciornea@uaic.ro (E.T.-C.); 3 Department of Pharmacognosy, Faculty of Pharmacy, “Grigore T. Popa” University of Medicine and Pharmacy, 16 University Street, 700115 Iasi, Romania; oana.cioanca@gmail.com

The authors wish to make the following correction to their paper [1]:

The number corresponding to the affiliation of Lucian Hritcu had been changed from:

Lucian Hritcu ^1,^*

to

Lucian Hritcu ^2,^*

The authors would like to apologize for any inconvenience caused to the readers by these changes. The changes do not affect the scientific results.
